# Combination OX40 agonism/CTLA-4 blockade with HER2 vaccination reverses T cell anergy and promotes survival in tumor-bearing mice

**DOI:** 10.1186/2051-1426-3-S2-P360

**Published:** 2015-11-04

**Authors:** Stefanie Linch, Melissa J Kasiewicz, Michael McNamara, Ian Hilgart-Martiszus, Mohammad Farhad, William Redmond

**Affiliations:** 1 Earle A. Chiles Research Institute/Providence Health and Services, Portland, OR, USA; 2 Earle A. Chiles Research Institute, Providence Cancer Center, Portland, OR, USA

## 

Immunotherapy is gathering momentum as a primary therapy for cancer patients. However, monotherapies have limited efficacy in improving outcomes and only benefit a subset of patients. Combination therapies targeting multiple pathways can augment an immune response to further improve survival. Here, we demonstrate that dual anti-OX40/anti-CTLA-4 immunotherapy generated a potent antigen-specific CD8 T cell response, enhancing expansion, effector function, and memory T cell persistence. Importantly, OX40 and CTLA-4 expression on CD8 T cells was critical to maximally promote their expansion following combination therapy. Animals treated with combination therapy and vaccination using anti-DEC-205-HER2 had significantly improved survival in a mammary carcinoma model. Vaccination with combination therapy uniquely restricted Th2-cytokine production by CD4 cells, relative to combination therapy alone, and enhanced IFNα production by CD8 and CD4 cells. We observed an increase in MIP-1α/CCL3, MIP-1β/CCL4, RANTES/CCL5, and GM-CSF production by CD8 and CD4 T cells following treatment. Furthermore, this therapy was associated with extensive tumor destruction and T cell infiltration into the tumor. Notably, vaccination with combination therapy reversed anergy and enhanced the expansion and function of CD8 T cells recognizing a tumor-associated antigen in a spontaneous model of prostate adenocarcinoma. Collectively, these data demonstrate that the addition of an anti-DEC-205-HER2 vaccine with combined anti-OX40/anti-CTLA-4 immunotherapy augmented anti-tumor CD8 T cell function, while limiting Th2 polarization in CD4 cells and improving overall survival.

